# Predictors of e-cigarette use among individuals with asthma: findings from a cross-sectional population-based study

**DOI:** 10.1007/s11739-025-04045-8

**Published:** 2025-07-12

**Authors:** Yusuff Adebayo Adebisi, Najim Z. Alshahrani

**Affiliations:** 1 College of Social Sciences, University of Glasgow, 40 Bute Gardens, Glasgow, G12 8RT UK; 2 Department of Family and Community Medicine, Faculty of Medicine, University of Jeddah, Jeddah, Saudi Arabia

**Keywords:** Asthma, E-cigarette use, Cigarette smoking, Vaping, Public health

## Abstract

The use of electronic cigarettes (e-cigarettes) among individuals with asthma is rising, yet limited evidence exists on predictors of use in this clinical population. Understanding factors associated with e-cigarette use may help inform public health interventions. In this study, we analysed data from 2671 individuals aged 16 years and older with doctor-diagnosed asthma, drawn from the 2017, 2018, 2019, and 2021 waves of the Scottish Health Survey. Current e-cigarette use was defined as self-reported use at the time of the survey. We used multivariable logistic regression to examine factors associated with current e-cigarette use among individuals with asthma, estimating adjusted odds ratios (aORs), 95% confidence intervals (CIs), and p values for age, sex, education, socio-economic deprivation, smoking status, alcohol consumption, and self-rated health. Overall, 193 participants (7.2%) reported current e-cigarette use. Current smokers had significantly higher odds of using e-cigarettes compared to never smokers (aOR: 38.9; 95% CI: 18.5–82.0; *p* < 0.001). Former smokers also had increased odds of e-cigarette use relative to never smokers (aOR: 32.0; 95% CI: 15.2–67.2; *p* < 0.001). Each one-category increase in age group (spanning approximately 10 years) was associated with a 23% reduction in the odds of current e-cigarette use (aOR: 0.77; 95% CI: 0.69–0.86; *p* < 0.001). Participants living in less deprived areas had 15% lower odds of current e-cigarette use for each one-quintile increase in the Scottish Index of Multiple Deprivation (aOR: 0.85; 95% CI: 0.74–0.97; *p* = 0.016). E-cigarette use was not significantly associated with sex, educational level, alcohol consumption, or self-rated health. These findings indicate that e-cigarette use among individuals with asthma is more prevalent among younger participants, those living in socioeconomically deprived areas, and those with a current or former smoking history. Further longitudinal research is needed to explore usage trajectories and respiratory health impacts in this clinical population.

## Introduction

Asthma is a chronic respiratory disease that affects over 300 million people globally and poses a substantial burden on both individuals and healthcare systems [1]. Characterised by airway inflammation, bronchial hyperresponsiveness, and variable airflow obstruction, asthma is exacerbated by environmental and behavioural triggers, including tobacco smoke [2, 3]. Smoking among individuals with asthma has been consistently associated with poor disease control, increased frequency and severity of exacerbations, faster decline in lung function, and reduced responsiveness to corticosteroid treatment [4, 5]. Consequently, smoking cessation is a key priority in the clinical management of asthma [4]. Despite this, some people with asthma continue to smoke [6–8], often due to the addictive nature of nicotine, socioeconomic disadvantage, psychological distress, and the limited success of conventional cessation strategies. 

Electronic cigarettes (e-cigarettes) have emerged over the last decade as a widely used alternative to conventional tobacco products. Globally, over 82 million people use e-cigarettes [9]. These devices deliver nicotine through aerosolised vapour without combustion, which has prompted debate regarding their potential role in smoking cessation and harm reduction [9]. Several randomised controlled trials and systematic reviews have demonstrated that e-cigarettes can be effective smoking cessation aids [10, 11]. Although evidence remains mixed, several studies have reported respiratory improvements in smokers with asthma who switched to e-cigarettes [12–15]. Notably, two studies found that people who use e-cigarettes experienced improvements in spirometry, airway hyperresponsiveness, and symptom control over a 12–24 month follow-up period [14, 15]. These findings have raised interest in the possible utility of e-cigarettes as a harm reduction tool for asthma patients who smoke, particularly for those who are unable or unwilling to quit using traditional methods [13]. However, concerns remain regarding long-term safety, dual use with combustible tobacco, and the broader implications of vaping on respiratory health, especially in people with pre-existing conditions like asthma.

Despite the ongoing debate, e-cigarette use among individuals with asthma is increasing [16]. Understanding who is more likely to use e-cigarettes within this group is essential for guiding public health policy, clinical practice, and communication strategies. While general population studies have identified factors such as younger age, lower socioeconomic position, and smoking history as key predictors of e-cigarette use in Scotland [17], limited evidence exists for people living with asthma. Given the dual burden of asthma and tobacco smoking, there is a need for targeted research to identify behavioural, demographic, and socioeconomic characteristics associated with e-cigarette use in this subpopulation. This could help clarify the profile of users, inform tailored smoking cessation interventions, and support more effective clinical and public health strategies. This study addresses this evidence gap by analysing data from the Scottish Health Survey (2017–2021) to examine predictors of current e-cigarette use among individuals with asthma. The findings aim to contribute to a nuanced understanding of e-cigarette use in a clinical population where the balance of risks and benefits may differ from the general public.

## Methods

### Data source and study design

This study used data from the 2017, 2018, 2019, and 2021 waves of the Scottish Health Survey (SHeS), a cross-sectional, nationally representative survey of individuals living in private households across Scotland. A total of 25,128 participants were present in the combined dataset, with annual contributions of 5300 in 2017, 6790 in 2018, 6881 in 2019, and 6157 in 2021. The 2020 SHeS data, affected by the pandemic and categorised as experimental statistics, was excluded due to its lack of comparability with other years [17]. The survey is designed to monitor the health and health-related behaviours of the Scottish population and inform public health policy. It employs a multistage stratified probability sampling method based on the postcode address file, ensuring geographical and demographic representativeness [18]. Given the repeated cross-sectional design of the study, different private households and individuals were interviewed each year [17, 18]. Data collection was conducted through in-home interviews using computer-assisted personal interviewing, with sensitive questions self-completed by participants. For this study, we combined the four available years to maximise statistical power and obtain a more stable estimate of associations.

### Study population

The analysis was restricted to individuals aged 16 years and above who reported a doctor diagnosis of asthma. Asthma status was determined using the variable ConDr, where participants who selected “Yes” were retained. Respondents who answered “No”, “Don’t know”, “Refused”, or had a routing error were excluded. This resulted in a subpopulation of 3255 individuals with asthma.

### Outcome variable: current e-cigarette use

The primary outcome was current e-cigarette use, as reported in the variable Ecigtot16. Participants were asked whether they currently used, had previously used, or had never used e-cigarettes or vaping devices. A binary outcome variable was created, where individuals who reported current use were coded as 1, and those who reported former or never use were coded as 0. To ensure valid outcome classification, we excluded participants with missing, refused, or inapplicable responses on e-cigarette use, resulting in a final analytic sample of 2671 individuals with complete data.

### Predictor variables

A range of variables were included as predictors, based on previous evidence linking them to e-cigarette use [17, 19–21]. Age was analysed in categories (16–24, 25–34, 35–44, 45–54, 55–64, and 65+ years). Sex was recorded as male or female. Educational attainment was recoded into three categories: higher education (degree or equivalent), secondary/college-level education, and lower or no qualifications. Socioeconomic position was assessed using the Scottish Index of Multiple Deprivation (SIMD), which ranks small areas across Scotland based on domains such as income, education, and health. SIMD was analysed in quintiles, from the most to the least deprived. Smoking status was grouped into never smokers, former smokers, and current smokers, based on respondents’ history and current use of cigarettes. Alcohol consumption over the past 12 months was recoded into three categories: frequent drinkers (weekly or more often), occasional drinkers (monthly or less), and non-drinkers. Self-rated general health was assessed using a standard survey item and categorised into “very good/good”, “fair”, and “bad/very bad”.

### Statistical analysis

Descriptive statistics were used to summarise the characteristics of the sample by current e-cigarette use status. Categorical variables were presented as frequencies and percentages. Differences between each predictor and current e-cigarette use were assessed using Chi-square tests.

To examine associations between potential predictors and current e-cigarette use, two sets of binary logistic regression models were fitted. First, crude odds ratios (ORs) and their 95% confidence intervals (CIs) were estimated in univariable models for each predictor. Second, a multivariable logistic regression model was used to estimate adjusted odds ratios (aORs) with 95% CIs, controlling for all covariates simultaneously. Variables included in the final adjusted model were: age group, sex, smoking status, educational attainment, alcohol consumption, self-rated general health, SIMD, and survey year. Based on likelihood ratio tests (LRTs), age group and SIMD were modelled as continuous variables to improve model fit. Model diagnostics were further conducted to assess fit and stability. Multicollinearity was evaluated using variance inflation factors (VIFs). Overall model fit was also assessed using the area under the receiver operating characteristic (ROC) curve (AUC), the Hosmer–Lemeshow goodness-of-fit test, and the link test for specification error.

To further aid interpretation, adjusted predicted probabilities of current e-cigarette use were estimated across age groups and smoking status categories using marginal effects analysis.

Three sensitivity analyses were conducted. First, individuals who reported current use of both cigarettes and e-cigarettes (i.e. dual users) were excluded to assess whether the main findings held when the outcome was restricted to exclusive e-cigarette use. Second, smoking status was recoded into a binary variable, combining never and former smokers into a single ‘non-current smoker’ group, to assess whether the results were robust to potential instability introduced by the small number of never smokers who reported e-cigarette use (*n* = 8). Third, orthogonal polynomial contrast tests were used to evaluate the appropriateness of treating age group and SIMD quintile as linear terms by testing for significant higher-order (non-linear) effects in the multivariable logistic regression model.

All analyses were conducted using Stata version 18, with statistical significance set at *p* < 0.05.

## Results

Table 1 presents the characteristics of individuals with asthma by current e-cigarette use status. Among the 2671 participants included in the analysis, 193 (7.2%) reported current e-cigarette use, while the majority (92.8%) were non-users (former or never users). Current use was most prevalent among individuals aged 25–34 and 45–54 years (both 22.8%). There was no significant difference in e-cigarette use by sex (*χ*^2^ = 0.04, *p* = 0.838), with similar proportions of males and females among users and non-users. Socioeconomic deprivation, measured using SIMD, showed a strong association with e-cigarette use (*χ*^2^ = 49.59, *p* < 0.001). Educational attainment also differed significantly between groups (*χ*^2^ = 21.75, *p* < 0.001). Over half (52.3%) of current e-cigarette users were also current cigarette smokers, and 43.5% were former smokers. In contrast, only 4.2% of current users were never smokers. Alcohol consumption in the past year was also significantly associated with e-cigarette use (*χ*^2^ = 11.72, *p* = 0.003). Non-drinkers represented a larger proportion of current users (28.5%) compared to non-users (19.0%). Self-rated health differed significantly by e-cigarette use status (*χ*^2^ = 12.02, *p* = 0.002).

**Table 1:** Characteristics of individuals with asthma by e-cigarette use status (Scottish Health Survey, 2017–2021)

Variable	Non-user (*n* = 2478)	Current e-cigarette user (*n* = 193)	Total (*n* = 2671)	*χ*^2^, *p* value
*Age group, n (%)*				*χ*^2^ = 22.03, *p* = 0.001
16–24	192 (7.8)	19 (9.8)	211 (7.9)	
25–34	389 (15.7)	44 (22.8)	433 (16.2)	
35–44	427 (17.2)	33 (17.1)	460 (17.2)	
45–54	403 (16.3)	44 (22.8)	447 (16.7)	
55–64	439 (17.7)	25 (13.0)	464 (17.4)	
65 +	628 (25.3)	28 (14.5)	656 (24.6)	
*Sex, n (%)*				*χ*^2^ = 0.04, *p* = 0.838
Male	983 (39.7)	78 (40.4)	1061 (39.7)	
Female	1495 (60.3)	115 (59.6)	1610 (60.3)	
*Deprivation, n (%)*				*χ*^2^ = 49.59, *p* < 0.001
Most deprived	426 (17.2)	65 (33.7)	491 (18.4)	
2	511 (20.6)	53 (27.5)	564 (21.1)	
3	495 (20.0)	31 (16.1)	526 (19.7)	
4	549 (22.2)	28 (14.5)	577 (21.6)	
Least deprived	497 (20.1)	16 (8.3)	513 (19.2)	
*Educational level, n (%)*				*χ*^2^ = 21.75, *p* < 0.001
Higher education (degree and above)	940 (37.9)	41 (21.2)	981 (36.7)	
Secondary/college	1210 (48.8)	117 (60.6)	1327 (49.7)	
Lower or no qualifications	328 (13.3)	35 (18.1)	363 (13.6)	
*Smoking status, n (%)*				*χ*^2^ = 245.80, *p* < 0.001
Never smoker	1493 (60.3)	8 (4.2)	1501 (56.2)	
Former smoker	588 (23.7)	84 (43.5)	672 (25.2)	
Current smoker	397 (16.0)	101 (52.3)	498 (18.6)	
*Alcohol consumption in the last 12 months, n (%)*				*χ*^2^ = 11.72, *p* = 0.003
Frequent drinker	1175 (47.4)	73 (37.8)	1248 (46.7)	
Occasional drinker	832 (33.6)	65 (33.7)	897 (33.6)	
Non-drinker	471 (19.0)	55 (28.5)	526 (19.7)	
*Self-rated health, n (%)*				*χ*^2^ = 12.02, *p* = 0.002
Very good/good	1506 (60.8)	95 (49.2)	1601 (59.9)	
Fair	610 (24.6)	55 (28.5)	665 (24.9)	
Bad/very bad	362 (14.6)	43 (22.3)	405 (15.2)	

Table 2 presents the crude and adjusted odds ratios (ORs) for factors associated with current e-cigarette use among individuals with asthma. Younger participants were significantly more likely to report current e-cigarette use, and the odds decreased progressively with each higher age category (aOR: 0.77; 95% CI: 0.69–0.86; *p* < 0.001). Similarly, individuals living in less deprived areas had reduced odds of current use compared to those in more deprived areas (aOR: 0.85; 95% CI: 0.74–0.97; *p* = 0.016). Smoking status was the strongest predictor. Compared to never smokers, former smokers had 32 times the odds of current e-cigarette use (aOR: 32.0; 95% CI: 15.2–67.2; *p* < 0.001), and current smokers had nearly 39 times the odds (aOR: 38.9; 95% CI: 18.5–82.0; *p* < 0.001). Although crude associations were observed for alcohol consumption, only borderline evidence of association remained after adjustment. No significant adjusted associations were observed for sex, educational level, or self-rated health.

**Table 2:** Crude and adjusted odds ratios for predictors of current e-cigarette use among individuals with asthma (Scottish Health Survey, 2017–2021)

Variable	Crude OR (95% CI), *p* value	Adjusted OR (95% CI), *p* value
*Age group (modelled as continuous)*	0.84 (0.77–0.92), *p* < 0.001	0.77 (0.69–0.86), *p* < 0.001
*Sex*		
Male	Reference	Reference
Female	0.97 (0.72–1.31), *p* = 0.838	1.06 (0.77–1.45), *p* = 0.741
*Deprivation (modelled as continuous)*	0.68 (0.61–0.76), *p* < 0.001	0.85 (0.74–0.97), *p* = 0.016
*Educational level*		
Higher education (degree and above)	0.41 (0.26–0.65), *p* < 0.001	0.98 (0.56–1.72), *p* = 0.951
Secondary/college	0.91 (0.61–1.35), *p* = 0.626	1.03 (0.66–1.62), *p* = 0.884
Lower or no qualifications	Reference	Reference
*Smoking status*		
Never smoker	Reference	Reference
Former smoker	26.7 (12.8–55.4), *p* < 0.001	32.0 (15.2–67.2), *p* < 0.001
Current smoker	47.5 (22.9–98.4), *p* < 0.001	38.9 (18.5–82.0), *p* < 0.001
*Alcohol consumption in the last 12 months*		
Frequent drinker	0.53 (0.37–0.77), *p* = 0.001	0.66 (0.43–1.00), *p* = 0.051
Occasional drinker	0.67 (0.46–0.97), *p* = 0.036	0.68 (0.45–1.04), *p* = 0.072
Non-drinker	Reference	Reference
*Self-rated health*		
Very good/good	Reference	Reference
Fair	1.43 (1.01–2.02), *p* = 0.043	0.99 (0.68–1.46), *p* = 0.980
Bad/very bad	1.88 (1.29–2.74), *p* = 0.001	0.99 (0.64–1.54), *p* = 0.986

Adjusted odds ratios (aORs) are derived from a multivariable logistic regression model that included all listed variables: age, sex, SIMD, educational level, smoking status, alcohol consumption, self-rated health and survey year

Reference categories are indicated

*OR* odds ratio, *CI* confidence interval

Model diagnostics indicated acceptable model performance and specification. The model showed strong discrimination, with an area under the ROC curve (AUC) of 0.84, indicating good ability to distinguish between current e-cigarette users and non-users. The model’s pseudo-*R*^2^ was 0.23 (*χ*^2^ = 314.07, *p* < 0.001). The Hosmer–Lemeshow goodness-of-fit test was borderline significant (*χ*^2^ = 15.78, *p* = 0.046), suggesting some deviation between observed and predicted probabilities in risk deciles, although this may reflect sensitivity to the large sample size. The link test showed no evidence of misspecification, with a significant _hat term (*p* = 0.013) and a non-significant _hatsq term (*p* = 0.336). Multicollinearity diagnostics using uncentered variance inflation factors (VIFs) revealed values ranging from 1.41 to 6.44, with a mean VIF of 3.10. All VIFs were below the commonly cited threshold of 10 for problematic collinearity [22], indicating that multicollinearity was not a concern and that the regression estimates are stable.

**Fig. 1:**
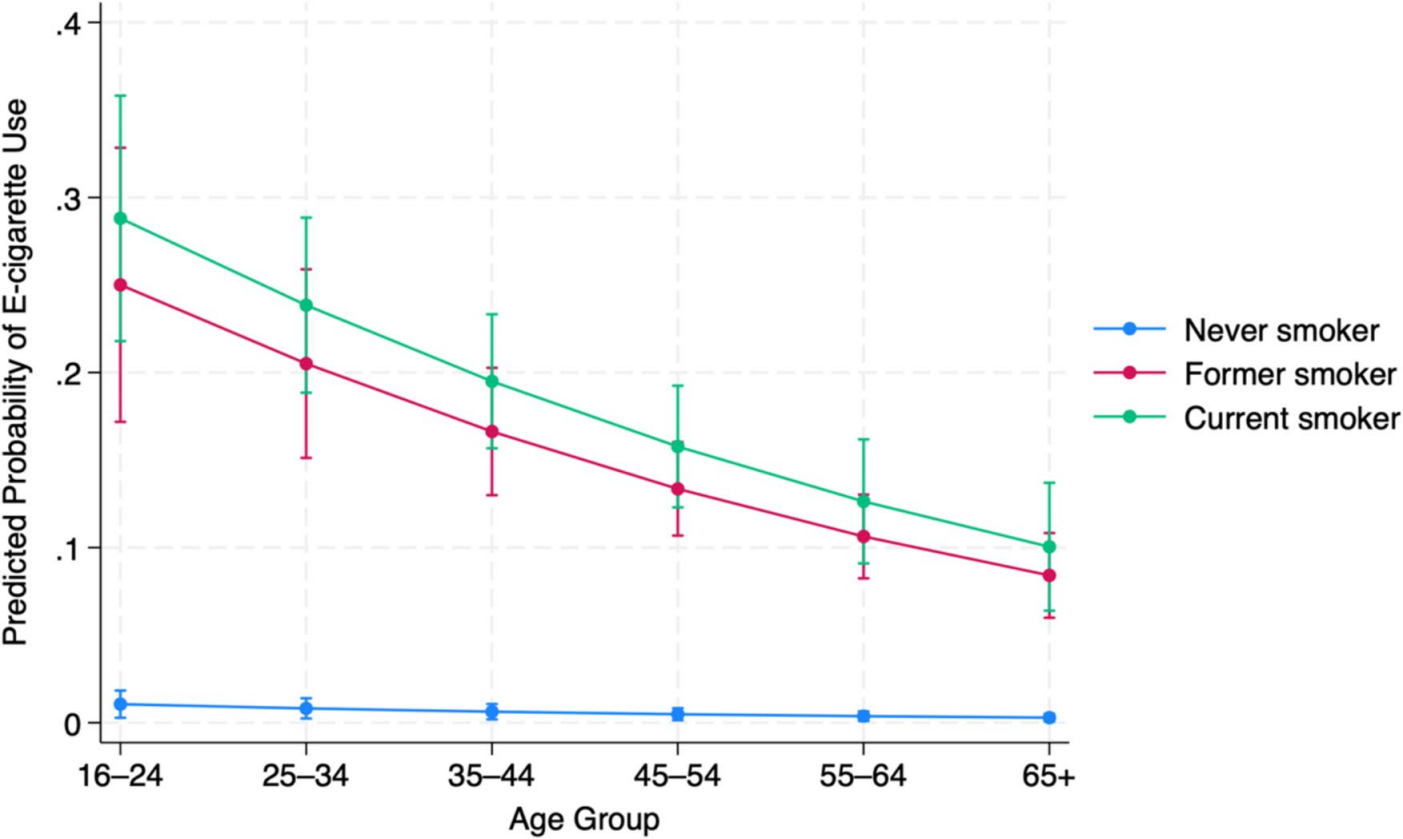
Adjusted predicted probability of current e-cigarette use by smoking status and age group among individuals with asthma (Scottish Health Survey, 2017–2021)

Figure 1 illustrates the adjusted predicted probabilities of current e-cigarette use across smoking status and age group among individuals with asthma. Across all age groups, current and former smokers consistently showed markedly higher probabilities of current e-cigarette use compared to never smokers. Among individuals aged 16–24, the predicted probability of current e-cigarette use was 28.8% for current smokers (95% CI: 21.8–35.8%), 25.0% for former smokers (95% CI: 17.2–32.8%), and 1.1% for never smokers (95% CI: 0.3–1.8%). These probabilities steadily declined with increasing age. For example, among individuals aged 45–54, the predicted probabilities were 15.8% (95% CI: 12.3–19.2%) for current smokers, 13.4% (95% CI: 10.7–16.0%) for former smokers, and 0.5% (95% CI: 0.1–0.8%) for never smokers. In the oldest age group (65 +), the probabilities dropped further to 10.0% for current smokers (95% CI: 6.4–13.7%), 8.4% for former smokers (95% CI: 6.0–10.8%), and 0.3% for never smokers (95% CI: 0.1–0.5%). While current and former smokers had significantly higher probabilities of e-cigarette use than never smokers at all ages, the differences between current and former smokers were narrower in older groups and not statistically significant.

### Sensitivity analyses

Three sensitivity analyses were conducted to test the robustness of the main findings.

First, individuals who reported dual use of cigarettes and e-cigarettes (*n* = 101) were excluded, limiting the outcome to exclusive e-cigarette use. The association between former smoking and exclusive e-cigarette use remained strong and statistically significant, with former smokers having over 40 times the odds of exclusive use compared to never smokers (aOR: 40.6; 95% CI: 18.8–87.7; *p* < 0.001). Younger age and residence in more socioeconomically deprived areas also remained independently associated with higher odds of exclusive e-cigarette use (aOR for age group: 0.62; 95% CI: 0.53–0.73; *p* < 0.001; aOR for SIMD quintile: 0.79; 95% CI: 0.66–0.95; *p* = 0.013). Other covariates, including sex, education, alcohol use, and self-rated health, remained non-significant. These results suggest the observed associations are not driven by dual users and are robust when restricted to exclusive e-cigarette use.

Second, smoking status was recoded into a binary variable, combining never and former smokers into a single ‘non-current smoker’ reference group. In this alternative specification, current smokers had over four times the odds of e-cigarette use compared to non-current smokers (aOR: 4.26; 95% CI: 3.06–5.94; *p* < 0.001). This finding supports the robustness of the primary results and demonstrates that the association between current smoking and e-cigarette use holds even when smoking status is simplified into a binary classification.

Third, orthogonal polynomial contrast tests were performed to assess the appropriateness of modelling age group and SIMD quintile as linear terms in the multivariable logistic regression model. For age group, only the linear trend was statistically significant (*χ*^2^ = 19.41, *p* < 0.001), while all higher-order terms (quadratic to quintic) were non-significant (*p* > 0.1). For SIMD, a modest but statistically significant linear component was observed (*χ*^2^ = 4.56, *p* = 0.033), and all higher-order terms were non-significant (*p* > 0.6). These findings support the decision to retain linear terms for both variables in the primary regression model.

## Discussion

This study provides important insights into the predictors of e-cigarette use among individuals with asthma in Scotland. The adjusted analysis revealed that younger age, residence in more socioeconomically deprived areas, and a history of cigarette smoking were the strongest predictors of current e-cigarette use in this population. Notably, age demonstrated an inverse relationship with use, with each increasing age category associated with a 23% reduction in the odds of using e-cigarettes. This age gradient aligns with broader population-level trends, suggesting that younger respondents are more likely to adopt e-cigarettes [17]. Socioeconomic deprivation also emerged as a significant correlate, with those living in more deprived areas showing higher odds of current e-cigarette use. This may reflect both greater tobacco-related harm and the potential appeal of e-cigarettes as a perceived harm reduction tool in resource-constrained settings where access to cessation support is limited [17, 23]. These patterns are consistent with frameworks on the social determinants of health, which emphasise how structural disadvantage shapes access to cessation resources and health behaviours [23]. They also align with behavioural models that highlight how perceived risk, opportunity, and access can influence uptake of alternative nicotine products [17, 23].

The most notable finding was the association between smoking status and e-cigarette use. Compared to never smokers, former smokers had 32 times the odds of using e-cigarettes, and current smokers had nearly 39 times the odds, highlighting smoking history as the dominant correlate of use. This pattern suggests that, among individuals with asthma, current e-cigarette use is concentrated among individuals with a current or past history of tobacco smoking. These findings support previous literature reporting that e-cigarettes are disproportionately used by people trying to quit smoking [24, 25], including those with asthma who may experience worsening respiratory outcomes from continued cigarette use [14, 15]. Interestingly, variables such as sex, educational attainment, and self-rated health were not significantly associated with e-cigarette use after adjusting for other covariates, indicating that smoking status and deprivation levels may overshadow the effects of these factors in this clinical group. These findings were robust to sensitivity analysis excluding dual users, which allowed us to focus specifically on exclusive e-cigarette users. In this restricted sample, the association between former smoking and exclusive e-cigarette use remained strong, reinforcing the interpretation that e-cigarettes may be adopted as an alternative to combustible tobacco following cessation. However, the motivation for continued use cannot be determined from these data.

The adjusted predicted probabilities provide further insight into how e-cigarette use varies across smoking status and age groups among individuals with asthma. The likelihood of current use was substantially higher among current and former smokers than among never smokers, with this pattern evident across all age groups. However, predicted use declined progressively with increasing age, suggesting that younger individuals, particularly those with a history of smoking, are more inclined toward e-cigarette use. For instance, among current smokers aged 16–24, nearly 3 in 10 were predicted to be current e-cigarette users, compared to just 1 in 100 among never smokers of the same age. This may reflect generational differences in attitudes toward e-cigarettes, including greater acceptance, perceived reduced harm, and familiarity with newer nicotine delivery technologies among younger people. In contrast, older individuals may be less receptive to e-cigarette use [26], potentially due to more established cessation behaviours, nicotine dependence level, misperceptions, or a reluctance to adopt unfamiliar alternatives.

Interestingly, the difference in predicted use between current and former smokers narrowed with age, possibly indicating a convergence in behaviour over time, where former smokers become less likely to maintain e-cigarette use as they grow older. These trends suggest that both smoking history and life stage play important roles in shaping e-cigarette use patterns in this clinical population. These trends suggest that both smoking history and life stage play important roles in shaping e-cigarette use patterns in this clinical population. Although the cross-sectional design precludes establishing the temporal sequence of smoking and e-cigarette use, it is unlikely that vaping preceded smoking in this population. Given the clinical risks of tobacco use in asthma and the very low prevalence of e-cigarette use among never smokers, it is more plausible that e-cigarettes are being adopted following cigarette use. The high uptake among former smokers further supports this interpretation, suggesting that some individuals may continue using e-cigarettes after quitting smoking.

These findings have several implications for public health policy and clinical practice. First, interventions to support smoking cessation in people with asthma should be sensitive to age and socioeconomic context. Younger people and those from more deprived backgrounds were more likely to report current e-cigarette use, which may reflect broader structural factors such as differential exposure to nicotine marketing, disparities in access to cessation support, and social patterning of tobacco-related behaviours. Second, given the strong association between smoking history and e-cigarette use, clinicians may consider engaging in informed, non-judgemental discussions with asthma patients who smoke or have smoked—addressing available cessation support, the potential risks and benefits of switching to e-cigarettes, and the importance of eventually quitting nicotine use altogether.

Some evidence suggests that switching from combustible tobacco to e-cigarettes may improve asthma control and quality of life without worsening lung function in the short term [27]. However, caution is warranted. While studies have reported harm reduction potential of e-cigarettes [28–32], their long-term safety in asthma populations remains uncertain. Therefore, public health messaging should remain balanced, acknowledging emerging evidence for smokers with asthma who cannot quit, while continuing to discourage uptake among never smokers and reinforcing the importance of evidence-based cessation strategies [33, 34]. Future research should explore the longitudinal impacts of e-cigarette use in asthma populations, particularly across different age and deprivation groups, to inform equitable and effective policy development.

A key strength of this study is its use of a large, nationally representative dataset spanning multiple years. By pooling data from four waves of the Scottish Health Survey (2017–2021) and applying rigorous statistical adjustment, the study offers robust and temporally stable estimates of associations between sociodemographic factors, smoking status, and current e-cigarette use. The stratified sampling design and high-quality data collection methods reduce the risk of selection and information bias, while the use of marginal effects to generate adjusted predicted probabilities provides a more intuitive understanding of usage patterns across age and smoking history. Additionally, the focus on individuals with asthma, a clinical subgroup often underrepresented in e-cigarette research, fills a critical evidence gap and offers valuable insights for targeted policy and clinical practice. Furthermore, diagnostic checks confirmed the robustness of the multivariable model, with no evidence of problematic multicollinearity across included predictors.

However, the study has several limitations. Its cross-sectional design precludes causal inference and limits the ability to determine temporal relationships between the predictors and e-cigarette use. Self-reported measures of asthma, smoking, and e-cigarette use are subject to recall and social desirability bias, which may affect the accuracy of responses. Furthermore, the analysis did not capture the frequency or intensity of e-cigarette use, dual use patterns, or motivations for vaping, which are important for understanding health implications. Finally, the exclusion of data from 2020, due to concerns about comparability, slightly reduced the overall sample size but does not impact the study’s primary objective of identifying predictors of e-cigarette use. In addition, the extremely low prevalence of e-cigarette use among never smokers may have contributed to inflated odds ratio estimates for former and current smokers due to sparse data bias. While the model was well specified and no separation was detected, these estimates should be interpreted with caution and in the context of wide confidence intervals. Additionally, although the dataset is nationally representative, selection bias may still be present due to differential non-response or exclusion of individuals without complete data, which could limit the generalisability of findings. As this is an exploratory analysis using secondary data, we cannot rule out potential residual confounding.

## Conclusion

This study provides important evidence on the correlates of current e-cigarette use among individual with asthma in Scotland. These findings highlight that younger age, residence in more socioeconomically deprived areas, and a history of cigarette smoking are key factors associated with e-cigarette use in this population. Notably, smoking status emerged as the strongest predictor, with both current and former smokers significantly more likely to report e-cigarette use compared to never smokers. Adjusted predicted probabilities further emphasised the influence of age and smoking history on usage patterns, with the highest likelihood of e-cigarette use observed among younger smokers. These insights underline the need for tailored cessation and harm reduction strategies that consider the unique profiles of individuals with asthma, particularly those who continue to smoke. While some individuals may be using e-cigarettes in an attempt to reduce or quit smoking, further longitudinal research is needed to understand the long-term safety, effectiveness, and role of e-cigarettes in asthma care. Public health messaging and clinical guidance should aim to support smoking cessation without inadvertently encouraging uptake of e-cigarettes among never smokers.

## Data Availability

To download the dataset used in the analyses, please visit the https://ukdataservice.ac.uk/find-data/browse/health/.

## Declarations

### Patient and public involvement

Patients and/or the public were not involved in the design, or conduct, or reporting, or dissemination plans of this research.
